# Supplementary material for: Bee Pollen and Bread as a Super-Food: A Comparative Review of Their Metabolome Composition and Quality Assessment in the Context of Best Recovery Conditions
Source: Molecules. 2023 Jan 11;28(2):715. doi: 10.3390/molecules28020715 (PMC9862147; doi:10.3390/molecules28020715)
Supplement: Supplementary file 1 [file molecules-28-00715-s001.zip › molecules-2139318-supplementary.pdf]

Supplementary Materials

Bee Pollen and Bread as a Super-Food: A Comparative Review of Their Metabolome Composition and Quality Assessment in the Context of Best Recovery Conditions

Mostafa H. Baky [1], Mostafa B. Abouelela [1], Kai Wang [2,*] and Mohamed A. Farag [3,*]

[1] Pharmacognosy Department, College of Pharmacy, Egyptian Russian University, Badr City P.O. Box 11829, Egypt
[2] Institute of Apicultural Research, Chinese Academy of Agricultural Sciences, Beijing P.O. Box 101101, China
[3] Pharmacognosy Department, College of Pharmacy, Cairo University, Kasr el Aini St., Cairo P.O. Box 11562, Egypt
* Correspondence: kaiwang628@gmail.com (K.W.); mohamed.farag@pharma.cu.edu.eg (M.A.F.)

Table S1. Flavonoids identified in bee bread and bee pollen

Sample/compounds	Isorhamnetin	Myricetin	Quercetin	Epicatechin	Naringenin	Hesperitin	Kaempferol	catechin	Apigenin	Luteolin	Tyrosol	Rutin	Naringenin	Ref.
Bee pollen sample	---	--	29.648 μg/g	--	21.92 μg/g	3.0 μg/g	7.8μg/g	--	--	--	--	--	--	[1]
Commercial Bee pollen	--	--	22.02 mg/kg	--	--	--	--	22.1 mg/kg		--	--	--	----	[2]
Indian bee pollen	--	--	3.14–15.94 mg/100 g	--	--	--	9.35 mg/100g	0.9–19.1 mg/100 g	0.4–3.0 mg/100 g	1.0–5.8 mg/100 g	--	4.8–24.8 mg/100 g	--	[3]
Bee pollen samles	--	20.4-244.7mg/100g	--	--	--	--	--	--	--	--	--	--	--	[4]
Bee bread samples	1227.9 mg/100 g	--	381.1–3918.1 mg/100g	--	--	--	112.94–2681.2mg/100g	--	--	21.9-3490.8 mg/100 g	--	--	--	
Rhododendron ponticum BP	--	3744.3 μg/100 g	--	1350.4 μg/100 g	--	--	--	1207.0 μg/100	--	--	1137.6 μg/1	--	--	[5]

								g			00 g			
Mixture of pollen samples	--	--	24.0–529.8 μg/g	--	--	--	--	--	--	--	--	156.2–955.7 μg/g	3.1–43.6 μg/g	[6]
Mixture of pollen samples	--	2220.70 μg/kg	7849.8 μg/kg	--	--	--	9870.7μg/kg	--	--	--	--	115,442.25 μg/kg	--	[7]

**Table S2. Phenolic acids reported in bee bread and bee pollen**											
Sample/ acids	Gallic	Caffeic	Ferulic	o-, *p*-coumaric	Cinnamic	Protocatechuic	Chlorogenic	2,5-dihydroxybenzoic	2-Hydroxy cinnamic	Ferulic	Ref.
Bee pollen sample	---	--	149.104 µg/g	36.688 µg/g	23.456 µg/g	--	--	--	--	--	[1]
Commercial Bee pollen	--	--	--	11.62 mg/kg	3.70 mg/kg	--	--	--	--	--	[2]
Indian bee pollen	--		--	--	--	--	--	--	--	--	[3]
Bee pollen samples	0.04-3.14 mg/100g	12.46-56.17 mg/100g	23.23–107.68 mg/100g	--	--	--	--	--	--	--	[4]
Bee bread samples	34.65 – 347.37 mg/100g	--	--	28.70–142.44 mg/100 g	--	166.61 mg/100g	3.89–36.09 mg/100g	2.69–35.09 mg/100g	--	--	
Mixture of pollen samples	3.0-32.3 µg/g	8.5-20.6 µg/g	--	--	--	--	--	--	43.4–179.9 µg/g	14.6 - 68.6 µg/g	[6]

Reference

1. Fanali, C.; Dugo, L.; Rocco, A. Nano-liquid chromatography in nutraceutical analysis: Determination of polyphenols in bee pollen. *Journal of Chromatography a* **2013,** *1313*, 270–274.
2. Toribio, L.; Arranz, S.; Ares, A. M.; Bernal, J. Polymeric stationary phases based on poly (butylene terephthalate) and poly (4-vinylpirydine) in the analysis of polyphenols using supercritical fluid chromatography. Application to bee pollen. *Journal of Chromatography A* **2018,** *1572*, 128–136.
3. Thakur, M.; Nanda, V. Screening of Indian bee pollen based on antioxidant properties and polyphenolic composition using UHPLC-DAD-MS/MS: A multivariate analysis and ANN based approach. *Food Research International* **2021,** *140*, 110041.
4. Bayram, N. E.; Gercek, Y. C.; Çelik, S.; Mayda, N.; Kostić, A. Ž.; Dramićanin, A. M.; Özkök, A. Phenolic and free amino acid profiles of bee bread and bee pollen with the same botanical origin–similarities and differences. *Arabian Journal of Chemistry* **2021,** *14*, 103004.
5. Ecem Bayram, N. Vitamin, mineral, polyphenol, amino acid profile of bee pollen from Rhododendron ponticum (source of “mad honey”): Nutritional and palynological approach. *Journal of Food Measurement and Characterization* **2021,** *15*, 2659–2666.
6. Kaškonienė, V.; Ruočkuvienė, G.; Kaškonas, P.; Akuneca, I.; Maruška, A. Chemometric analysis of bee pollen based on volatile and phenolic compound compositions and antioxidant properties. *Food Analytical Methods* **2015,** *8*, 1150–1163.
7. Gercek, Y. C.; Celik, S.; Bayram, S. Screening of Plant Pollen Sources, Polyphenolic Compounds, Fatty Acids and Antioxidant/Antimicrobial Activity from Bee Pollen. *Molecules* **2022,** *27*, 117.
